# Biomarkers identified for prostate cancer patients through genome-scale screening

**DOI:** 10.18632/oncotarget.20739

**Published:** 2017-09-08

**Authors:** Lei-Yun Wang, Jia-Jia Cui, Tao Zhu, Wei-Hua Shao, Yi Zhao, Sai Wang, Yu-Peng Zhang, Ji-Chu Wu, Le Zhang

**Affiliations:** ^1^ Department of Clinical Pharmacology, XiangYa Hospital, Central South University, Changsha 410008, P.R. China; ^2^ Institute of Clinical Pharmacology, Central South University, Hunan Key Laboratory of Pharmacogenetics, Changsha 410078, P.R. China; ^3^ Department of Neurology, XiangYa Hospital, Central South University, Changsha 410008, P.R. China; ^4^ Department of Cardiovascular, Central Hospital of ShaoYang, ShaoYang 422000, P.R. China

**Keywords:** prostate cancer, biomarker screening, genome-scale, prognosis, gene expression

## Abstract

Prostate cancer is a threat to men and usually occurs in aged males. Though prostate specific antigen level and Gleason score are utilized for evaluation of the prostate cancer in clinic, the biomarkers for this malignancy have not been widely recognized. Furthermore, the outcome varies across individuals receiving comparable treatment regimens and the underlying mechanism is still unclear. We supposed that genetic feature may be responsible for, at least in part, this process and conducted a two-cohort study to compare the genetic difference in tumorous and normal tissues of prostate cancer patients. The Gene Expression Omnibus dataset were used and a total of 41 genes were found significantly differently expressed in tumor tissues as compared with normal prostate tissues. Four genes (SPOCK3, SPON1, PTN and TGFB3) were selected for further evaluation after Gene Ontology analysis, Kyoto Encyclopedia of Genes and Genomes pathway analysis and clinical association analysis. MIR1908 was also found decreased expression level in prostate cancer whose target genes were found expressing in both prostate tumor and normal tissues. These results indicated that these potential biomarkers deserve attention in prostate cancer patients and the underlying mechanism should be further investigated.

## INTRODUCTION

Prostate cancer is still a serious threat to men in the world, with a top 2 ranked mortality rate in males, second to that of lung cancer [1, 2]. Despite the wide application of radical prostatectomy, radiation therapy and androgen deprivation therapy in clinic [3], treatment outcome varies significantly across patients and is unpredictable [4]. Although prostate specific antigen (PSA) level and Gleason score were used for screening and evaluation of prostate cancer [5, 6], the genetic feature, critical for diagnosis and prognosis for prostate cancer patients, is still not widely recognized [7, 8]. Genome-scale screening is necessary to facilitate the understanding about the inner cause and progression of prostate cancer.

Genome-scale microarrays are used in cancer study as a powerful technology for years. Its high-throughput screening ability renders it a more ideal platform than traditional methods for researchers [9]. Based on microarray analysis, many genes were found related to prostate cancer, such as PIM1, AMACR, H3K27me3, and IL-15 [10–13]. However, poor reproducibility of results is still a potential problem and a comprehensive study integrating microarray data generated from different labs is necessary [14].

We utilized six independent genome-scale research datasets of prostate cancer patients and performed a two-stage study to search and verify the candidate biomarkers for this disease. A total of 41 genes were founded with consistent lower expression level in tumor tissues than in normal tissues in our discovery stage research. Four genes (SPOCK3, SPON1, PTN and TGFB3) and a micro-RNA (MIR1908) were selected for exploring their potential roles in the progression of prostate cancer. We found that SPOCK3, SPON1, PTN and TGFB3 were significantly correlated with the progression-free survival (PFS) status of prostate cancer patients. The target genes of MIR1908 were predicted and were found transcribed actively in prostate tumor tissues and normal prostate tissues. Our study indicated that these five potential biomarker genes may play important roles in prostate cancer and underlying mechanisms could be further studied in the future.

## RESULTS

### Screening for candidate biomarkers in prostate tumorous and normal tissues

**Figure 1:**
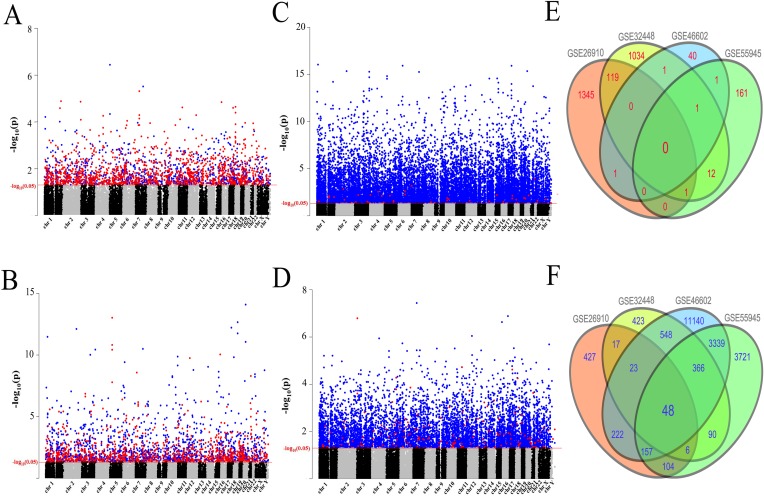
Search for candidate biomarkers for prostate cancer patients **(A-D)** Manhattan plots of up-regulated probes (marked in red) and down-regulated probes (marked in blue). The red line indicated the p-value was 0.05. (A) 1371 up-regulated probes and 972 down-regulated probes in GSE26910. (B) 1080 up-regulated probes and 1472 down-regulated probes in GSE32448. (C) 42 up-regulated probes and 15119 down-regulated probes in GSE46602. (D) 167 up-regulated probes and 7432 down-regulated probes in GSE55945. **(E-F)** Venn plots of intersection parts of these probes in each dataset. (E) Venn plot for up-regulated probes. (F) Venn plot for down-regulated probes.

We chose four GEO datasets: GSE26910, GSE32448, GSE46602 and GSE55945 as our discovery-cohort to identify the difference of gene expression-profile between prostate tumor tissues and normal prostate tissues (Supplementary Table 1). A total of 94 tumor samples and 63 normal prostate samples were included in. GSE32448 contained 40 pairs of T-N tissue from Rockville and the other three datasets consisted of unmatched tumorous and normal tissues. The clinical information of patients was listed in Supplementary Table 2. We first explored the profile of differentially expressed genes among these four datasets and probes were defined as our measurement index. 1006 decreased expression level probes and 1466 increased expression level probes showing significantly different expression level were found in GSE26910. 1522 decreased expression level probes and 1168 increased expression level probes were considered as significantly differentially expressed probes in GSE32448. 15844 decreased expression level probes and 44 increased expression level probes in GSE46602, 7832 decreased expression level probes and 175 increased expression level probes in GSE55945 were also identified (Figure 1A-1D). All these probes were divided into two groups depending on if they were up-regulated or down-regulated. The intersection of two groups were regarded as candidate biomarkers in this step (Figure 1E-1F). The detail expression scenarios of these candidates in each dataset were shown in Supplementary Figure 1A-1D (Supplementary Figure 1). There were 48 candidates in the down-regulated group and 0 candidate in the up-regulated group after our rigorous screening, all of which were converted to corresponding gene symbols in the next step.

### Functional analysis of candidate biomarker genes

**Figure 2:**
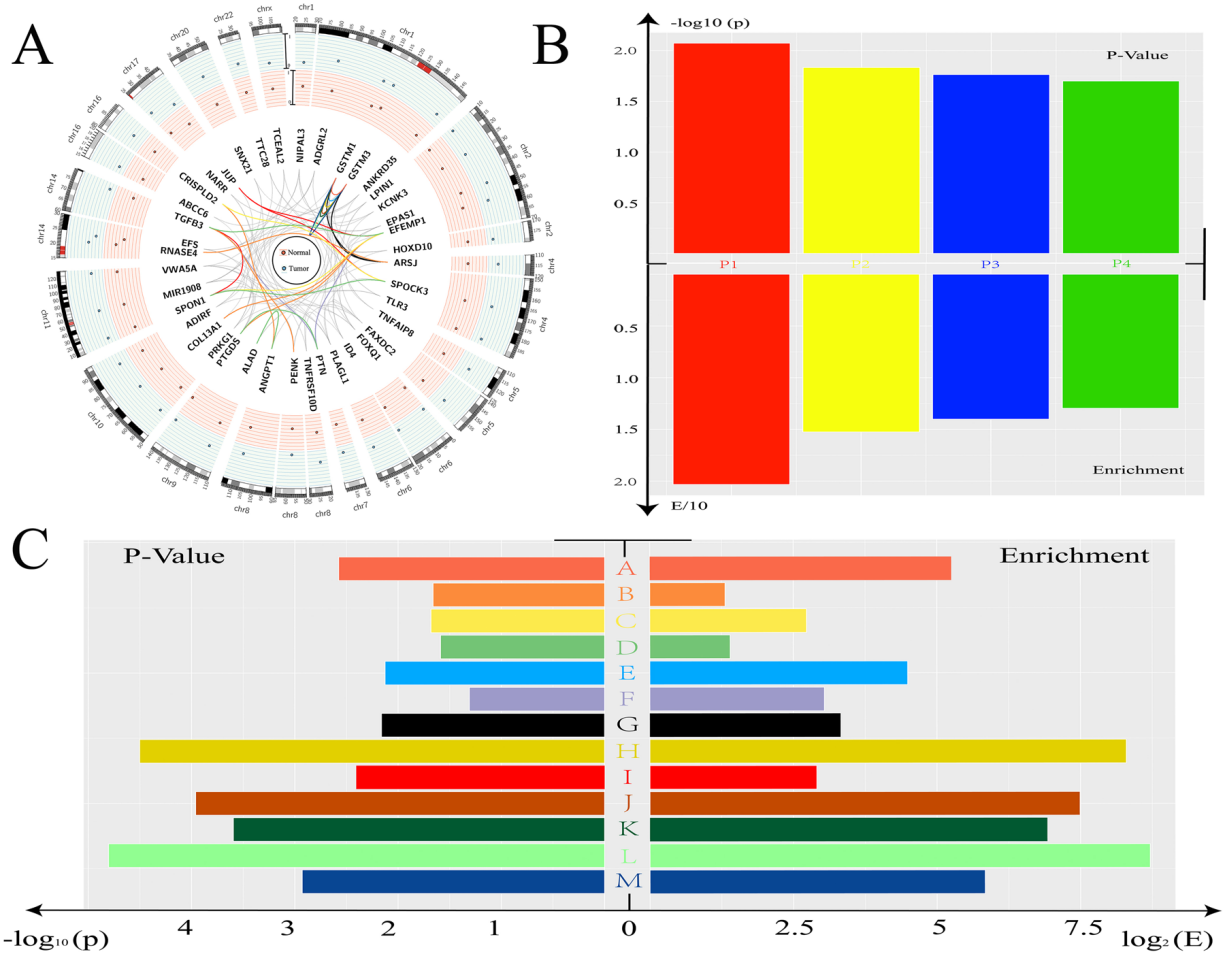
Function analysis for candidate biomarker genes **(A)** Circos plot showed all the genes found in previous stage. The location and expression level in Tumor/Normal tissue was indicated. The color of line was depended the GO analysis results listed in Figure 2C. **(B)** The p-value and enrichment-value was showed. P1: Glutathione metabolism. P2: Drug metabolism - cytochrome P450. P3: Metabolism of xenobiotics by cytochrome P450. P4: Chemical carcinogenesis. **(C)** The results of GO analysis were showed. A: glutathione transferase activity. B: extracellular region. C. proteinaceous extracellular matrix. D: extracellular space. E: glutathione metabolic process. F: growth factor activity. G: metabolic process. H: nitrobenzene metabolic process. I: extracellular matrix. J: xenobiotic catabolic process. K: glutathione binding. L: cellular detoxification of nitrogen compound. M: glutathione derivative biosynthetic process.

In order to explore the roles of these candidates, we first searched the known relationship between these genes and prostate cancer in PubMed. As mentioned above, a total of 41 genes were identified in the previous stage. We listed all the genes that were studied before in prostate cancer among our results (Supplementary Table 3). The expression level of candidate genes in our discovery cohort and their chromosomal locations were presented in Figure 2A.

Gene Ontology (GO) function cluster analysis and Kyoto Encyclopedia of Genes and Genomes (KEGG) pathway analysis were used for further research. KEGG pathway analysis indicated that some candidates involved in Glutathione metabolism related pathway and Drug metabolism related pathway. Of note, Chemical carcinogenesis related pathway was significantly enriched according to the p-value in our analysis (Figure 2B), indicating that these candidate genes may play important roles in the process of cancer. Enriched GO terms indicated that these candidate genes played important roles in metabolic process and enzyme related function such as nitrobenzene metabolic process and glutathione derivative biosynthetic process (Figure 2C). We presented all these terms in this figure and the related genes in Figure 2A were indicated with lines of different colors.

### Biomarker genes’ expression level may indicate the prognosis for prostate cancer patients

Based on our screening criteria, all candidates showed lower levels in prostate cancer tissues than in normal prostate tissues and most of them were enriched in certain terms as indicated by GO analysis. This phenomenon is interesting and we then used an independent cohort to explore the relationship between these potential biomarkers’ expression levels and prognosis in clinic. Data of 436 prostate cancer patients from TCGA database were used in this section, the detail clinical information of patients was listed in Table 1.

**Table 1:** Clinic information of prostate cancer patients enrolled in progression-free-survival analysis

	SPOCK3low	SPOCK3high	p-value	SPON1low	SPON1high	p-value	PTNlow	PTNhigh	p-value	TGFB3low	TGFB3high	p-value
	Age			Age			Age			Age		
≤65	150	156	0.915	165	141	0.988	129	177	0.051	149	157	0.085
>65	63	67		70	60		68	62		75	55	
Gleason score				Gleason score			Gleason score			Gleason score		
6~7	109	165	<0.001	136	138	0.02	98	176	<0.001	126	148	0.003
8~10	104	68		99	63		99	63		98	64	
PSA value				PSA value			PSA value			PSA value		
>0.1	142	171	0.028	160	153	0.146	131	182	0.073	154	159	0.541
≤0.1	50	35		51	34		45	40		45	40	
others	21	17		24	14		21	17		25	13	
Pathology stage				Pathology stage			Pathology stage			Pathology stage		
T1-T2	74	105	0.01	93	86	0.511	60	119	<0.001	78	101	0.005
T3-T4	136	116		139	113		136	116		144	108	
others	3	2		3	2		1	4		2	3	

^*^Others means that related information were missing in the dataset.

**Figure 3:**
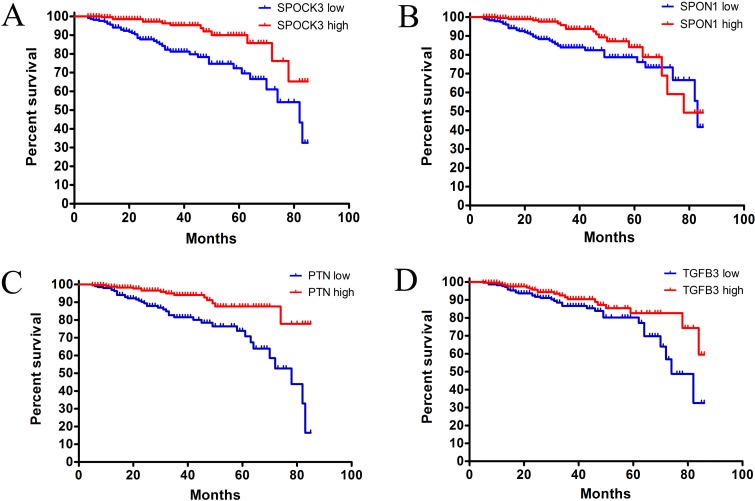
Progression-free-survival curve for potential biomarkers **(A)** The progression-free-survival curve for SPOCK3 high level group and SPOCK3 low level group. (p-value < 0.0001, hazard ratio: 3.345. 95% CI: 1.787-6.261) **(B)** The progression-free-survival curve for SPON1 high level group and SPON1 low level group. (p-value = 0.020, hazard ratio: 1.963. 95% CI: 1.100-3.506) **(C)** The progression-free-survival curve for PTN high level group and PTN low level group. (p-value < 0.0001, hazard ratio: 3.336. 95% CI: 1.833-6.073) **(D)** The progression-free-survival curve for TGFB3 high level group and TGFB3 low level group. (p-value < 0.046, hazard ratio: 1.754. 95% CI: 1.003-3.068).

We found that SPOCK3 and SPON1 were significantly associated with prostate cancer patients’ PFS. Patients with lower expression level of SPOCK3 showed worse PFS than those with higher SPOCK3 level (Figure 3A, p-value < 0.0001, HR = 3.345, 95% CI: 1.787 - 6.261). Patients with lower level of SPON1 had shorter PFS than patients with higher level of SPON1 (Figure 3B, p-value = 0.02, HR = 1.963, 95% CI: 1.100 - 3.506). Function of PTN and TGFB3 in prostate cancer patients were previously investigated, our related results were shown in Figure 3C-3D. The relative expression levels of these four genes were shown in Supplementary Figure 2-5.

These results indicated that SPOCK3, SPON1, PTN and TGFB3 may be chosen as potential prognostic biomarkers for prostate cancer patients in clinic.

### Low expression level of MIR1908 was found in prostate cancer patients

**Figure 4:**
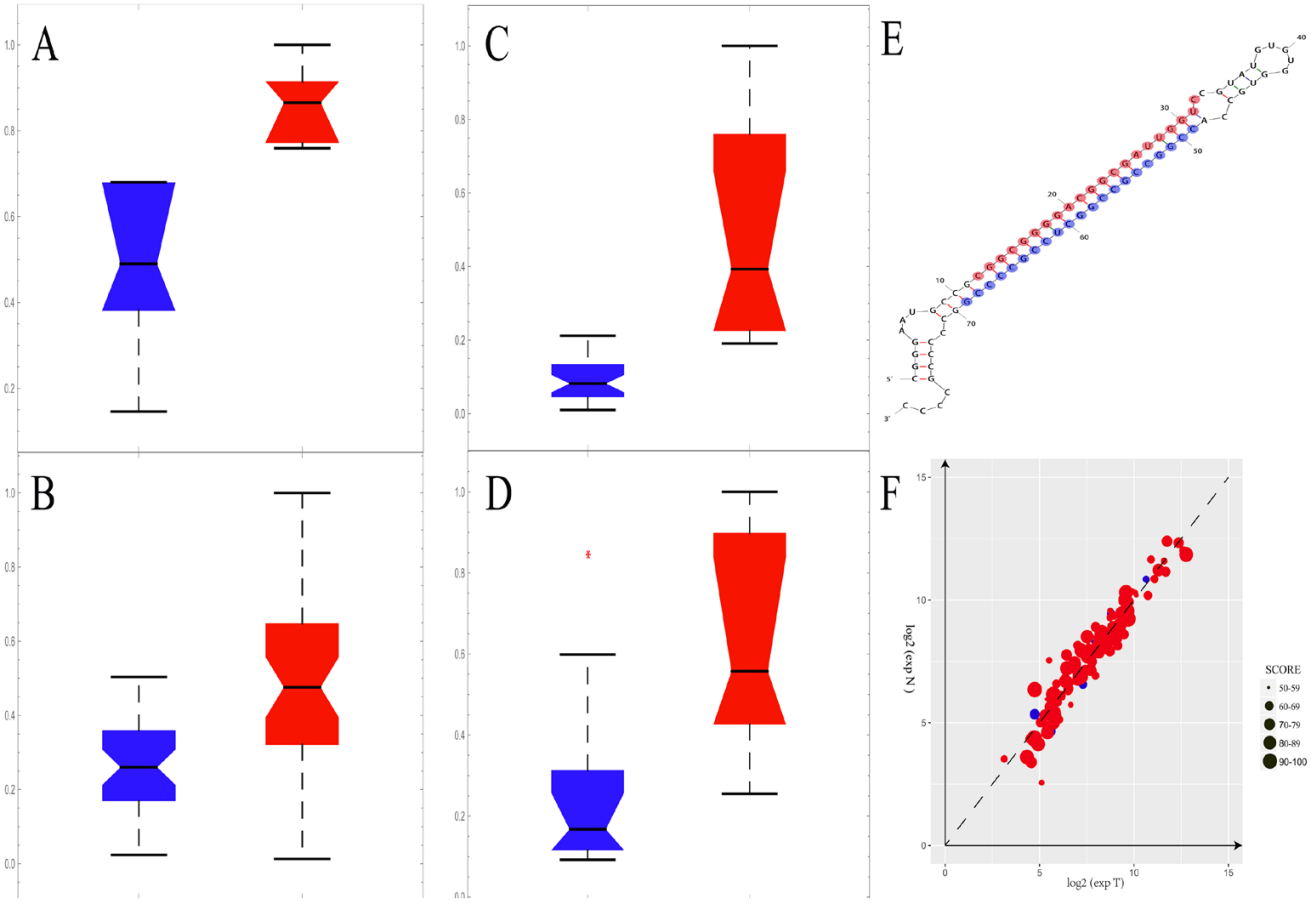
The MIR1908 may play an important role in prostate cancer patients **(A-D)** The expression level of MIR1908 was remarkably down-regulated in tumor tissue (blue) than normal (red) in the discovery stage. The red asterisk means outliers. (A) The expression level of MIR1908 in GSE26910. (B) The expression level of MIR1908 in GSE32448. (C) The expression level of MIR1908 in GSE GSE46602. (D) The expression level of MIR1908 in GSE55945. **(E)** The structure of MIR1908. The red bases indicated the 5P of this micro RNA and the blue bases indicated the 3P of this micro RNA. **(F)** The expression level of target Genes of MIR1908. The size of dot depended on the binding-score witch predicted by miRDB.

MIR1908 showed lower level in prostate tumor tissues in our discovery cohort and the distribution plot were provided (Figure 4A-4D). To our knowledge, the role of this microRNA in prostate cancer has not been reported. The structure of miR1908 was shown (Figure 4E) and its potential targets were predicted (Supplementary Table 4). We found that many target genes of this microRNA showed expression in both prostate normal tissues and tumor tissues (Figure 4F). These results indicated that underlying mechanisms about how this microRNA regulates its targets could be further studied.

### A validation cohort for confirming results in prostate cancer patients

**Figure 5:**
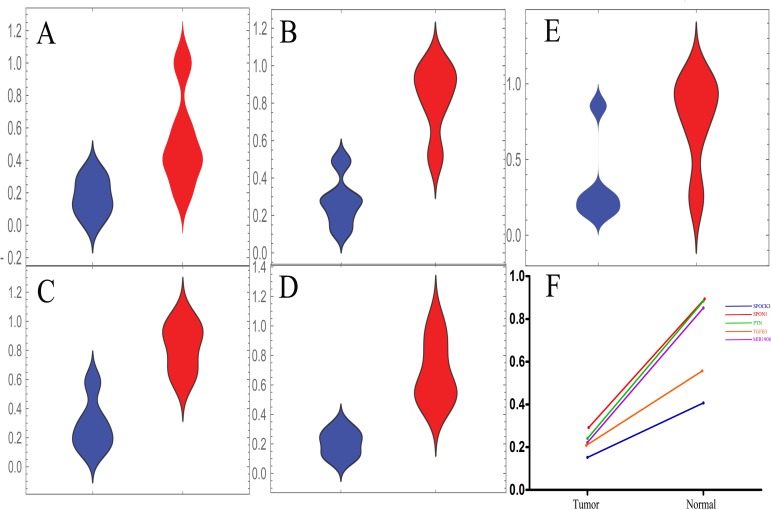
The validation stage for expression level of these five candidate biomarkers **(A-E)** The distribution plots of these biomarkers in the validation cohort. The tumor tissue group was marked in blue while normal was red. (A) The distribution status of SPOCK3 in the validation stage. (B) The distribution status of SPON1 in the validation stage. (C) The distribution status of PTN in the validation stage. (D) The distribution status of TGFB3 in the validation stage. (E) The distribution status of MIR1908 in the validation stage. **(F)** The median line values of distribution plots were calculated and the difference between tumor group and normal group was presented.

SPOCK3, SPON1 and MIR1908 were found to be potential biomarkers in our discovery stage. We further used a validation cohort in this section to ensure reliability of these results. PTN and TGFB3 were considered as control biomarkers as previously reported. A dataset with 5 paired tumor-normal tissues of prostate patients examined in the same platform were included in this stage. This dataset was published in an independent study (Supplementary Figure 6). Consistent with results from the discovery cohorts, all these potential biomarkers were downregulated in tumor tissues (Figure 5A-5E). Another two datasets generated from a different platform were included in this stage, too. A consistent result was observed (Supplementary Figure 7-8). These results indicated that SPOCK3, SPON1, MIR1908, PTN and TGFB3 may be treated as biomarkers for prostate cancer patients.

## DISCUSSION

We conducted a genome-scale research to seek candidate biomarker genes of prostate cancer patients. The datasets of microarray were utilized in this study for it's high-throughput capability [15]. We considered genes with a consistent expression trend in the four discovery datasets as potential biomarkers and 41 genes were picked out finally. There were 24 genes that had been reported correlating with prostate cancer, among which TGFB3 [16], PTN [17], ID4 [18] were widely studied. These results proved that our methods were valid in another aspect. To our knowledge, a panel of genes, including SPOCK3 and SPON1, were found downregulated significantly in prostate cancer patients for the first time. It was interesting to note that SPOCK3, SPON1, PTN and TGFB3 played important roles, as suggested by function enrichment analysis. Downregulation of these three genes in prostate cancer patients may affect extracellular margin and space as well as growth factor activity. GSTM1, GSTM3 and ABCC6 were metabolism related genes whose abnormal regulation may contribute to prostate cancer progression.

To investigate the prognostic significance of these genes in patients with prostate cancer, we chose an independent cohort to explore the association between them and PFS of prostate cancer patients. These results demonstrated that SPOCK3, SPON1 were correlated with PFS of prostate cancer patients in this cohort. PTN and TGFB3 were also found to influence the PFS of patients with prostate cancer. However, GSTM1, GSTM3 and ABCC6 showed no significant effects on prostate patients’ PFS (data not shown).

SPOCK3 (SPARC/osteonectin, cwcv and kazal like domains proteoglycan 3), also known as testican3, is a member of novel calcium-binding proteoglycan proteins family. Research indicated that the protein encoded by SPOCK3 might inhibit the activity of membrane-type matrix metalloproteinases [19] and was identified as a suppressor of tumor invasion [20]. SPON1 (spondin-1), also known as Vascular smooth muscle cell growth-promoting factor (VSGP), was found to be an inhibitor of angiogenesis [21]. The micro-vessel density of tumor may be affected by SPON1 according to a previous study [21]. PTN (Pleiotrophin) could restrain the differentiation of epithelial cells *in vivo* [17] and TGFB3 (Transforming growth factor-β 3) was shown to have tumor-suppressing function [16]. We found that MIR1908, which was found as a proliferation suppressor in NSCLC, [22], showed a lower expression mode in tumor tissues than in normal tissues.

There are some limitations in this study. First, we focused on screening of candidate biomarkers and the molecular functions of these biomarkers were not included in this study. Instead, the related Gene Ontology (GO) function analysis and Kyoto Encyclopedia of Genes and Genomes (KEGG) pathway analysis were carried out and some possible mechanisms of these five biomarkers which we focused on were discussed in the discussion section. Moreover, the platforms of our validation stage were not exactly as same as those of the discovery stage and the sample size of this study was limited. However, we successfully validated the candidate biomarkers from our discovery stage despite the different platforms and these results were consistent in all datasets generated from different labs, suggesting the reliability of our conclusion.

In summary, we conducted a multiple-cohort research to seek the candidate biomarker genes for prostate cancer patients in a genome-wide scale. Forty-one candidates were all down-regulated in the first cohort of them, SPOCK3, SPON1, PTN and TGFB3 were associated with the prognosis of prostate cancer patients. Besides, MIR1908 was a potential biomarker for prostate cancer patients according to our result. The underlying mechanism would be investigated in the future.

## MATERIALS AND METHODS

### Datasets of prostate tumor/normal tissue

Four GEO datasets (GSE26910, GSE32448, GSE46602 and GSE55945) of 94 tumor samples and 63 normal prostate samples were used as discovery cohort and three independent datasets (GSE17906, GSE6919, GSE38241) of 78 tumor samples and 79 normal prostate samples were used as validation cohort in this study (GEO, https://www.ncbi.nlm.nih.gov/geo/). Five datasets shared a same platform namely Affymetrix Human Genome U133 Plus 2.0 Array while GSE6919 and GSE38241 were performed in Affymetrix Human Genome U95 Version 2 Array and Agilent-014850 Whole Human Genome Microarray (4×44K G4112F) respectively. An independent dataset included 436 prostate cancer patients with evaluated PFS status were obtain from the UCSC Cancer Browser (https://genome-cancer.ucsc.edu/) database performed in IlluminaHiSeq_RNASeqV2 platform (Supplementary Table 1-2).

### Pre-process of data

We downloaded data files with probe values (.CEL files) from the GEO database. The raw data was read and pre-processed by AFFY package of R [23, 24]. Mas5 algorithm combined with detection calls of multiple Perfect-Match (PM) and Mismatch (MM) probes were utilized in the process of background correction, quantile normalization as well as calculation of the expression level of each probe [25].

### Candidate biomarkers screening

We performed t-test to evaluate the statistical significance of gene expression difference between tumor tissues and normal tissues [26]. A set of significant differential expression level probes (p-value < 0.05) were divided into tumor high expression group (HE) and tumor low expression group (LE). Intersection of HE or LE in the four discovery datasets was picked out as candidate biomarkers. Cluster analysis of these biomarkers in each dataset were performed afterward. The t-test analysis and presentation of our results were performed by basic package and gplots package of R respectively.

### Function analysis biomarkers screening

We annotated candidate biomarkers and performed GO function cluster analysis as well as KEGG pathway analysis by Annotation, Visualization, and Integrated Discovery (DAVID, https://david.ncifcrf.gov/) database [27]. Circos software and ggplot2 package of R were used for results presentation [28].

### MicroRNA structure and targets prediction

We predicted targets of MIR1908 by online miRDB software (http://www.mirdb.org/) [29]. The secondary structure of MIR1908 were predicted by M-Fold software provided online (http://unafold.rna.albany.edu/) [30].

### Statistical analysis

SPSS software (version 18.0, SPSS, Chicago, ILand), Mathematica software (version 10.0, Mathematica, Chicago, Champaign) and GraphPad Prism (version 5, GraphPad Software Inc, San Diego, CA) were utilized in this study for statistical analysis and results presentation. Student's t-tests were used in comparing the difference between two groups. Kaplan–Meier analysis were utilized to analyze the PFS of two groups and Log-rank tests were used to compare the difference. Results with p <0.05 were considered as statistically significant.

## SUPPLEMENTARY MATERIALS FIGURES AND TABLES


