# Supplementary material for: Biomarkers identified for prostate cancer patients through genome-scale screening
Source: Oncotarget. 2017 Sep 8;8(54):92055–63. doi: 10.18632/oncotarget.20739 (PMC5696163; doi:10.18632/oncotarget.20739)
Supplement: Supplementary file 1 [file oncotarget-08-92055-s001.pdf]

Biomarkers identified for prostate cancer patients through genome-scale screening

SUPPLEMENTARY MATERIALS

REFERENCES

1. Planche A, Bacac M, Provero P, Fusco C, Delorenzi M, Stehle JC, Stamenkovic I. Identification of prognostic molecular features in the reactive stroma of human breast and prostate cancer. PLoS One. 2011; 6: e18640. https://doi.org/10.1371/journal.pone.0018640.
2. Derosa CA, Furusato B, Shaheduzzaman S, Srikantan V, Wang Z, Chen Y, Seifert M, Ravindranath L, Young D, Nau M, Dobi A, Werner T, McLeod DG, et al. Elevated osteonectin/SPARC expression in primary prostate cancer predicts metastatic progression. Prostate Cancer Prostatic Dis. 2012; 15: 150-6. https://doi.org/10.1038/pcan.2011.61.
3. Mortensen MM, Hoyer S, Lynnerup AS, Orntoft TF, Sorensen KD, Borre M, Dyrskjot L. Expression profiling of prostate cancer tissue delineates genes associated with recurrence after prostatectomy. Sci Rep. 2015; 5: 16018. https://doi.org/10.1038/srep16018.
4. Arredouani MS, Lu B, Bhasin M, Eljanne M, Yue W, Mosquera JM, Bubley GJ, Li V, Rubin MA, Libermann TA, Sanda MG. Identification of the transcription factor single-minded homologue 2 as a potential biomarker and immunotherapy target in prostate cancer. Clin Cancer Res. 2009; 15: 5794-802. https://doi.org/10.1158/1078-0432.ccr-09-0911.
5. Pascal LE, Goo YA, Vencio RZ, Page LS, Chambers AA, Liebeskind ES, Takayama TK, True LD, Liu AY. Gene expression down-regulation in CD90+ prostate tumor-associated stromal cells involves potential organ-specific genes. BMC Cancer. 2009; 9: 317. https://doi.org/10.1186/1471-2407-9-317.
6. Chandran UR, Ma C, Dhir R, Bisceglia M, Lyons-Weiler M, Liang W, Michalopoulos G, Becich M, Monzon FA. Gene expression profiles of prostate cancer reveal involvement of multiple molecular pathways in the metastatic process. BMC Cancer. 2007; 7: 64. https://doi.org/10.1186/1471-2407-7-64.
7. Aryee MJ, Liu W, Engelmann JC, Nuhn P, Gurel M, Haffner MC, Esopi D, Irizarry RA, Getzenberg RH, Nelson WG, Luo J, Xu J, Isaacs WB, et al. DNA methylation alterations exhibit intraindividual stability and interindividual heterogeneity in prostate cancer metastases. Sci Transl Med. 2013; 5: 169ra10. https://doi.org/10.1126/scitranslmed.3005211.

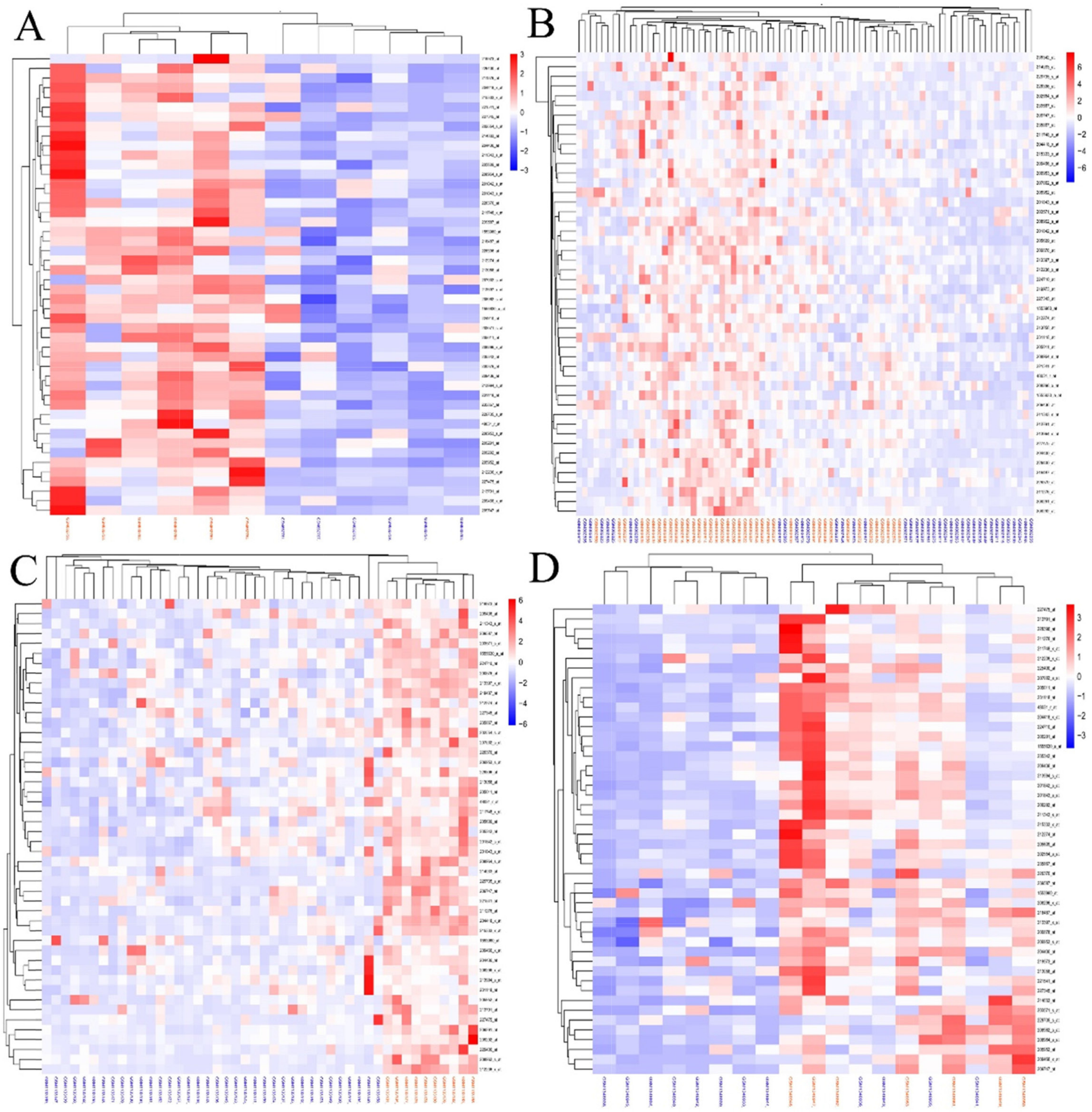

Supplementary Figure 1: Heatmap of probes expression level in discovery stage based on un-supervised clustering. (A) GSE26910. **(B)** GSE32448. **(C)** GSE46602. **(D)** GSE55945. Tumor samples were marked in blue and normal was red.

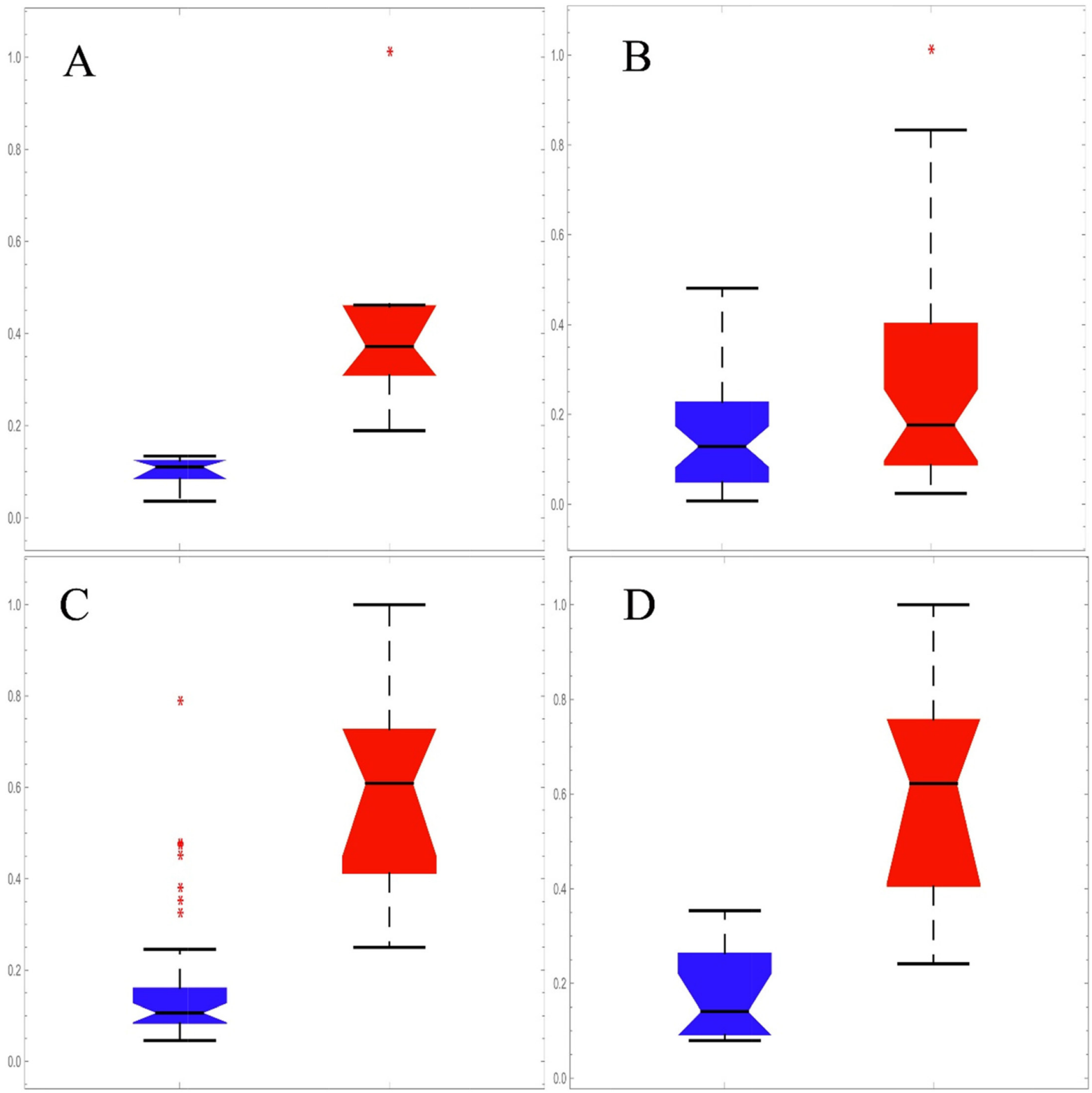

Supplementary Figure 2: The expression level of SPOCK3 was remarkably down-regulated in tumor tissue (blue) than normal (red) in the discovery stage. The red asterisk means outliers. **(A)** The expression level of SPOCK3 in GSE26910. **(B)** The expression level of SPOCK3 in GSE32448. **(C)** The expression level of SPOCK3 in GSE GSE46602. **(D)** The expression level of SPOCK3 in GSE55945.

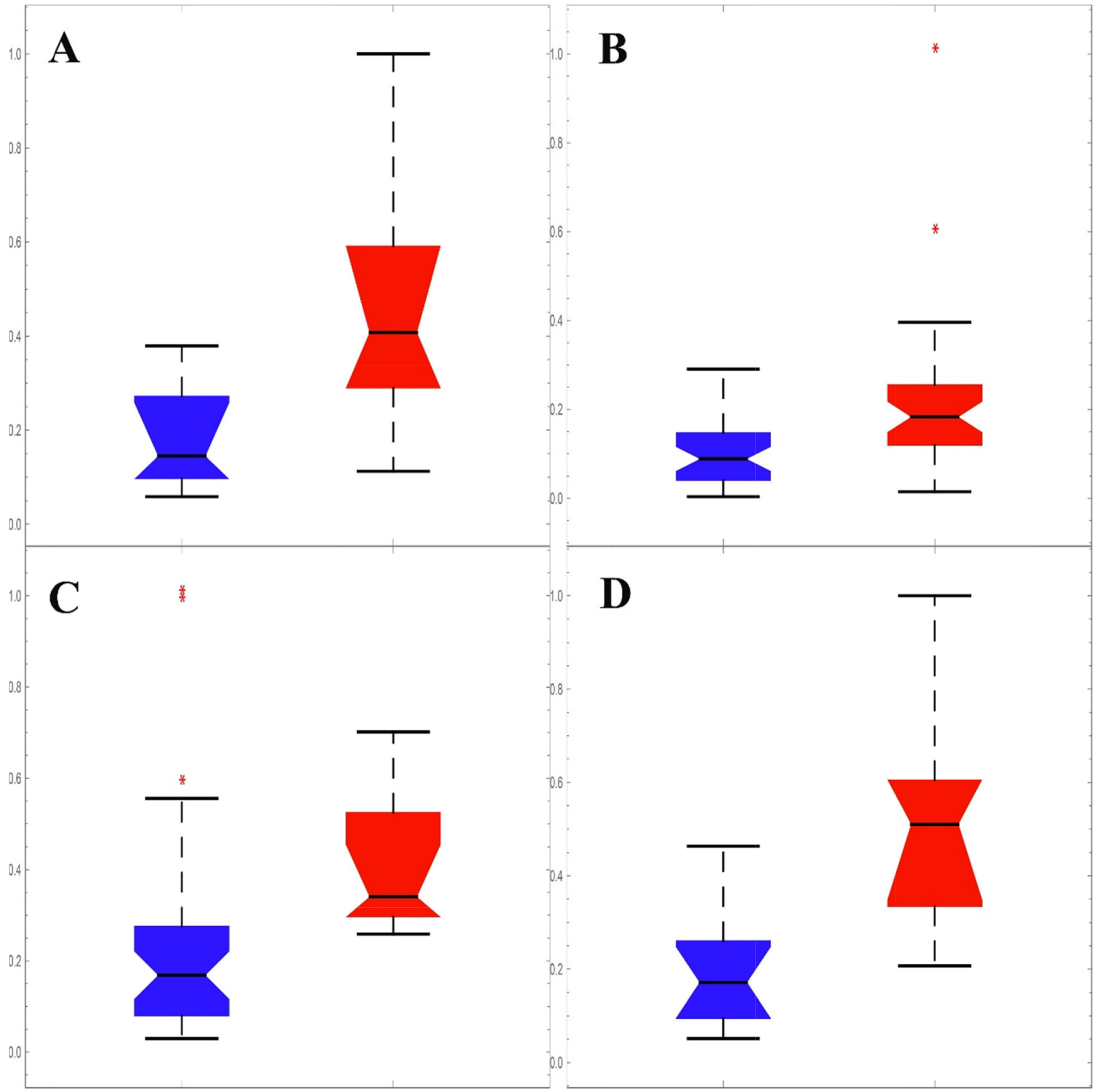

Supplementary Figure 3: The expression level of SPON1 was remarkably down-regulated in tumor tissue (blue) than normal (red) in the discovery stage. The red asterisk means outliers. **(A)** The expression level of SPON1 in GSE26910. **(B)** The expression level of SPON1 in GSE32448. **(C)** The expression level of SPON1 in GSE GSE46602. **(D)** The expression level of SPON1 in GSE55945.

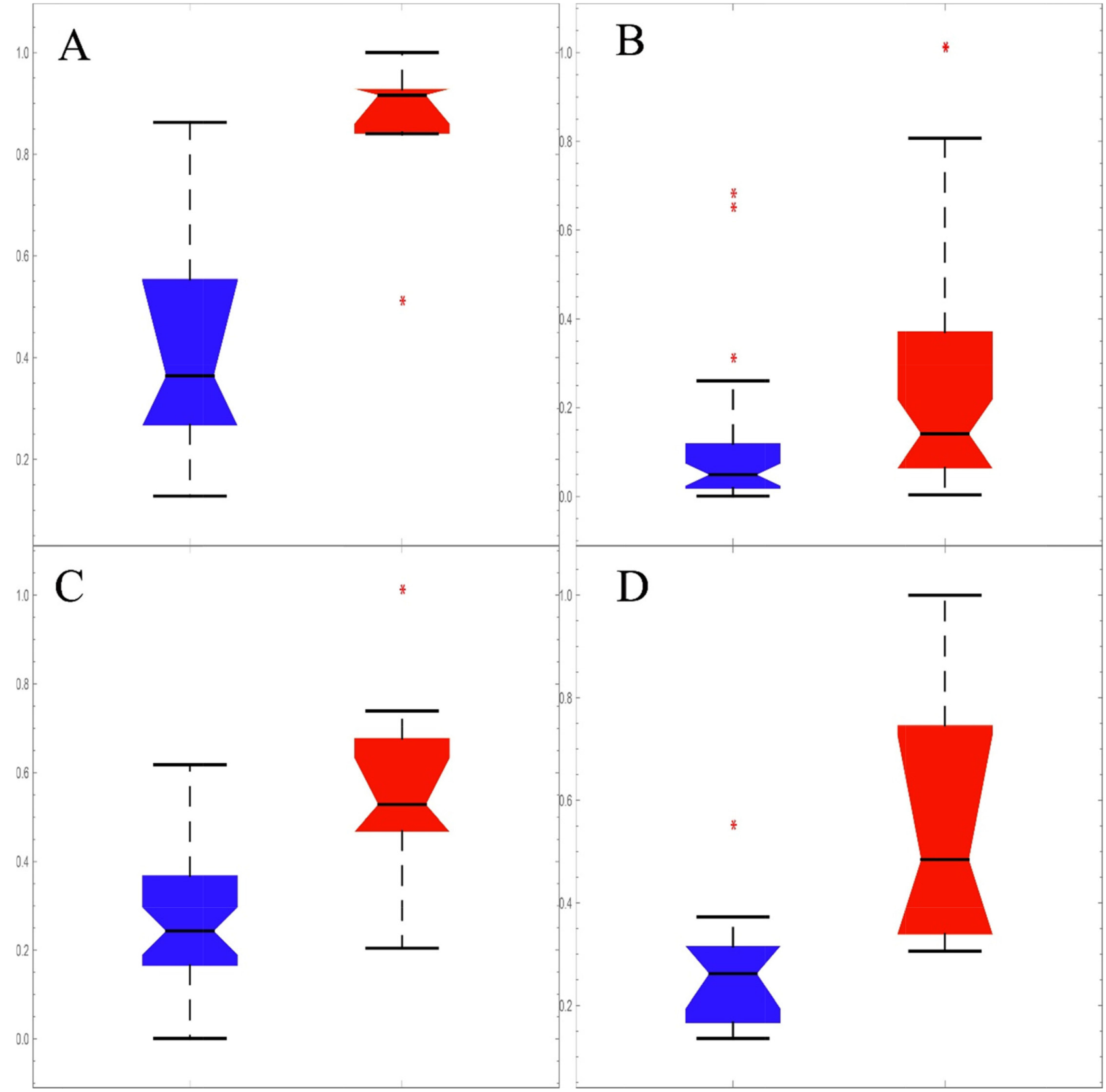

Supplementary Figure 4: The expression level of PTN was remarkably down-regulated in tumor tissue (blue) than normal (red) in the discovery stage. The red asterisk means outliers. **(A)** The expression level of PTN in GSE26910. **(B)** The expression level of PTN in GSE32448. **(C)** The expression level of PTN in GSE GSE46602. **(D)** The expression level of PTN in GSE55945.

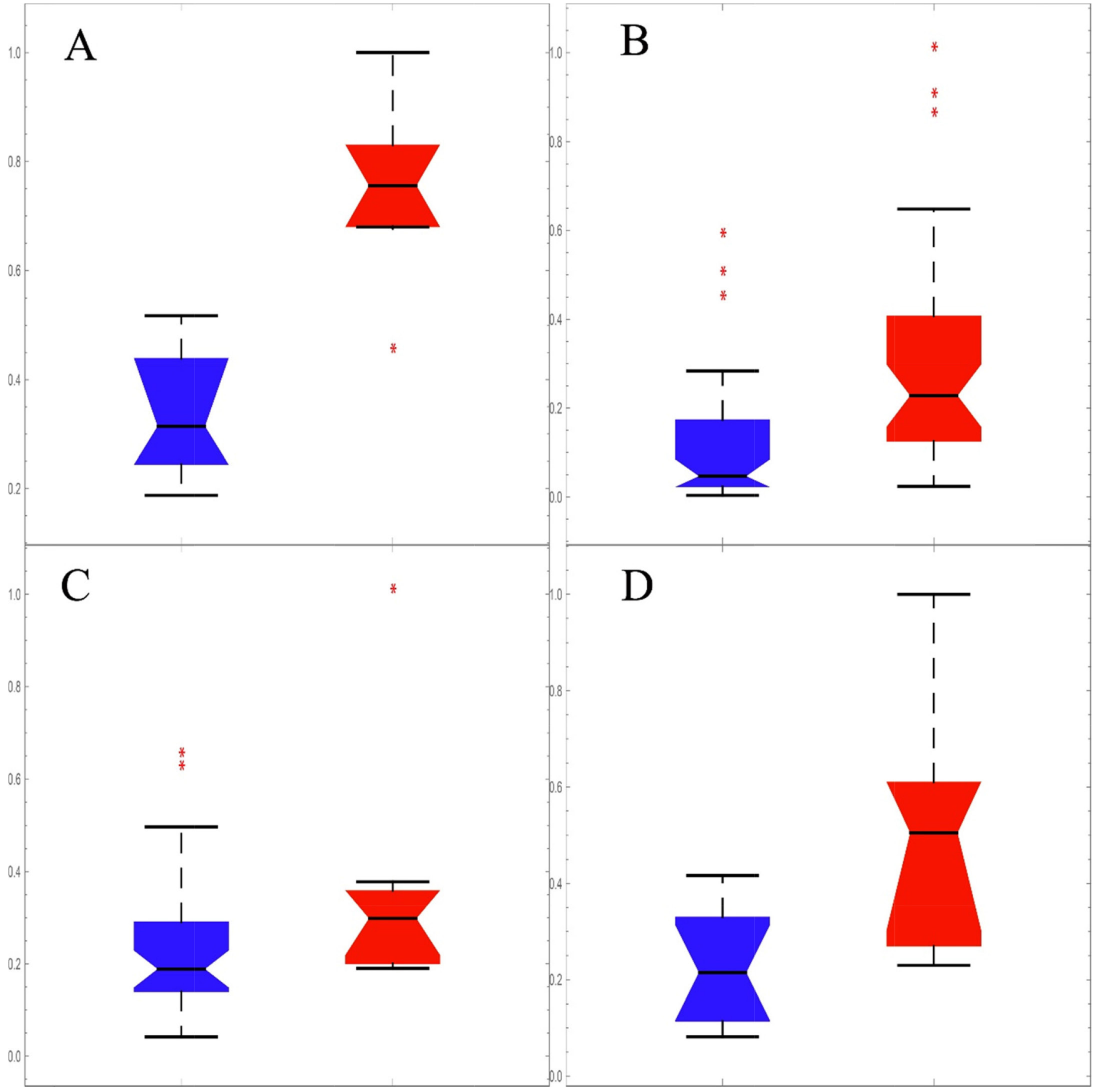

Supplementary Figure 5: The expression level of TGFB3 was remarkably down-regulated in tumor tissue (blue) than normal (red) in the discovery stage. The red asterisk means outliers. **(A)** The expression level of TGFB3 in GSE26910. **(B)** The expression level of TGFB3 in GSE32448. **(C)** The expression level of TGFB3 in GSE GSE46602. **(D)** The expression level of TGFB3 in GSE55945.

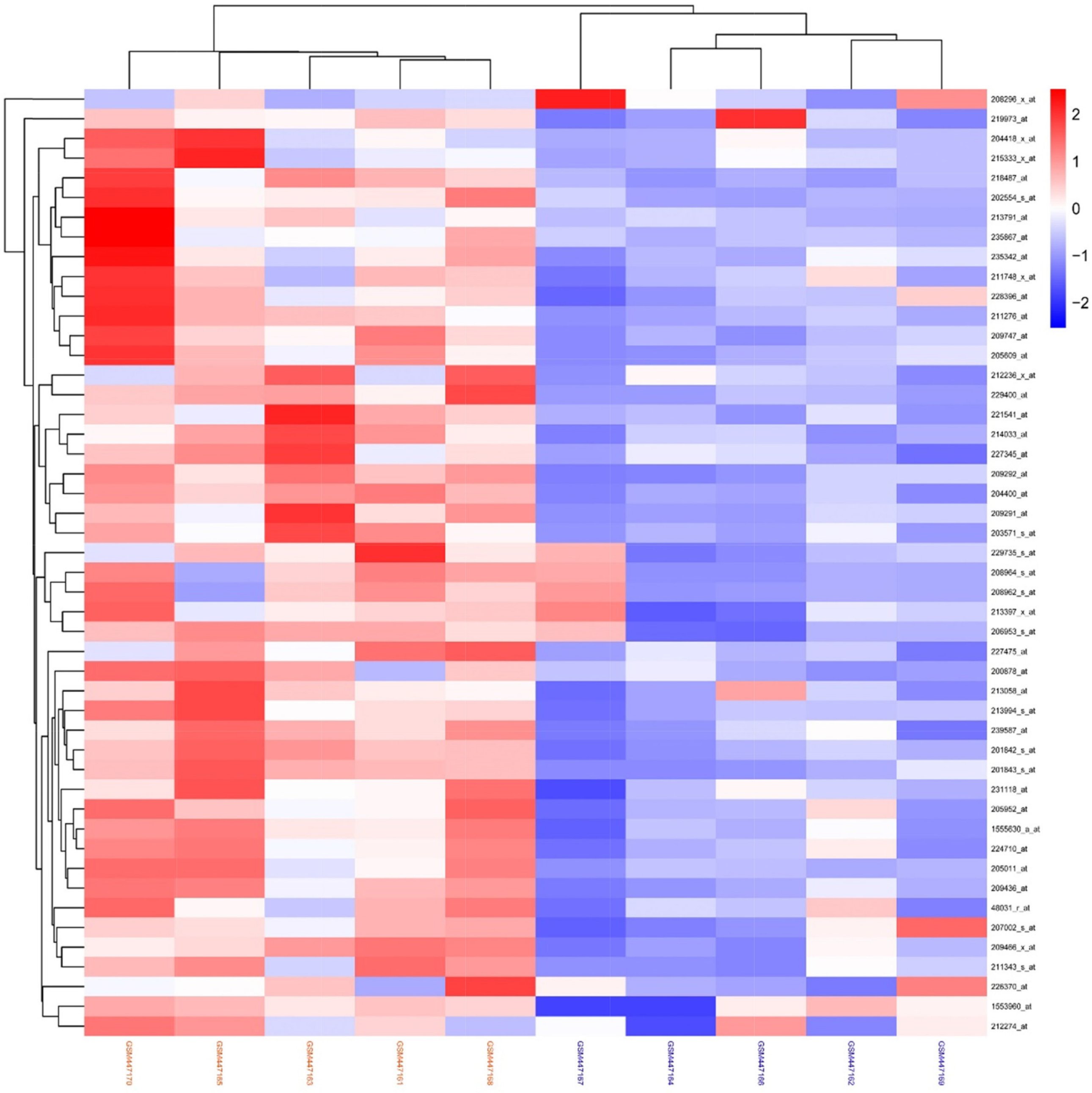

Supplementary Figure 6: Heatmap of probes expression level in validation stage based on un-supervised clustering. The dataset GSE17906 was used in this stage. Tumor samples were marked in blue and normal was red.

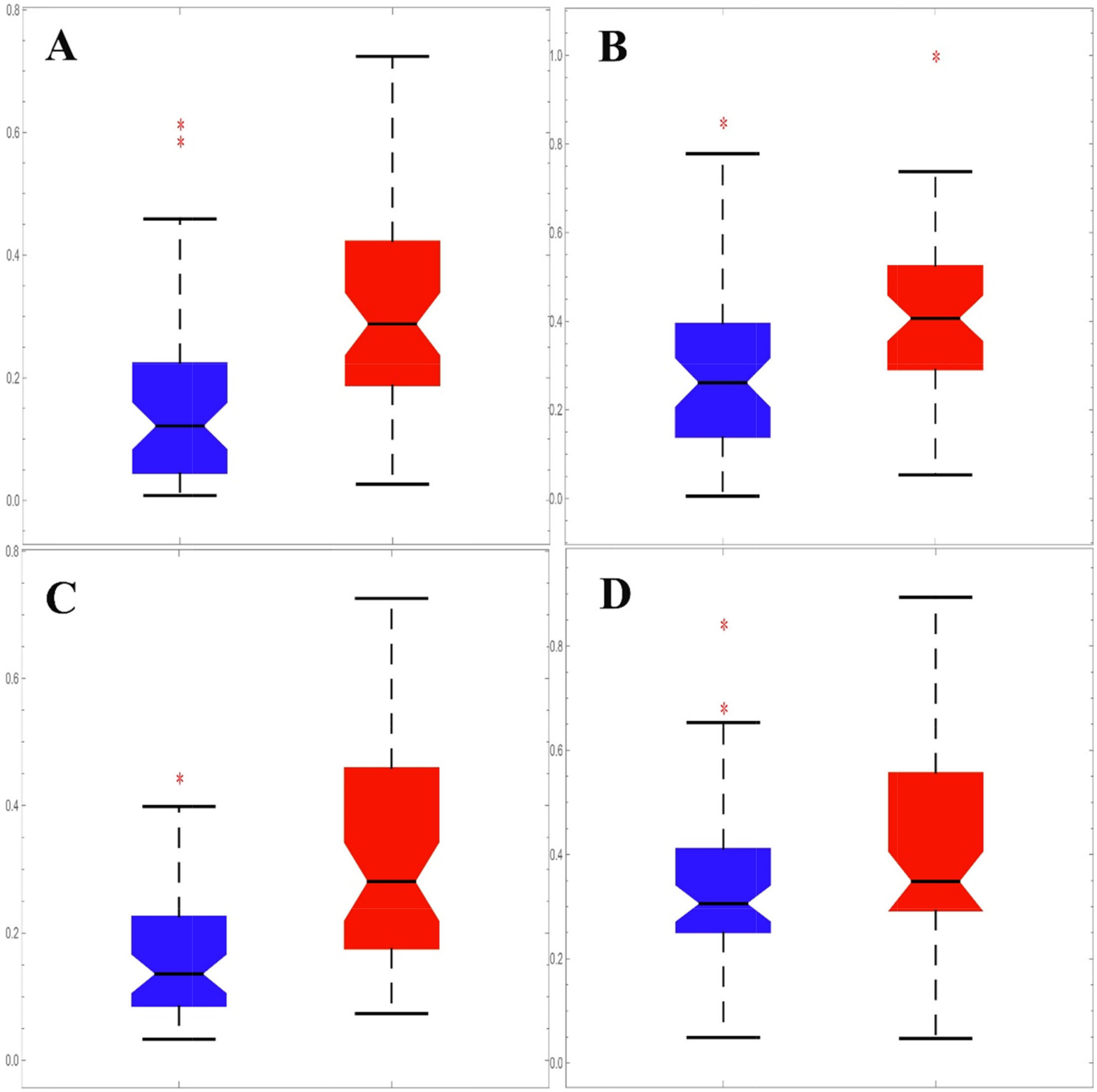

Supplementary Figure 7: The expression level of four important candidate biomarkers in tumor tissue (blue) and normal (red) was consistent with discovery stage (validation dataset GSE6919). **(A)** The expression level of SPOCK3 in GSE6919. **(B)** The expression level of PTN in GSE6919. **(C)** The expression level of TGFB3 in GSE6919. **(D)** The expression level of MIR1908 in GSE6919.

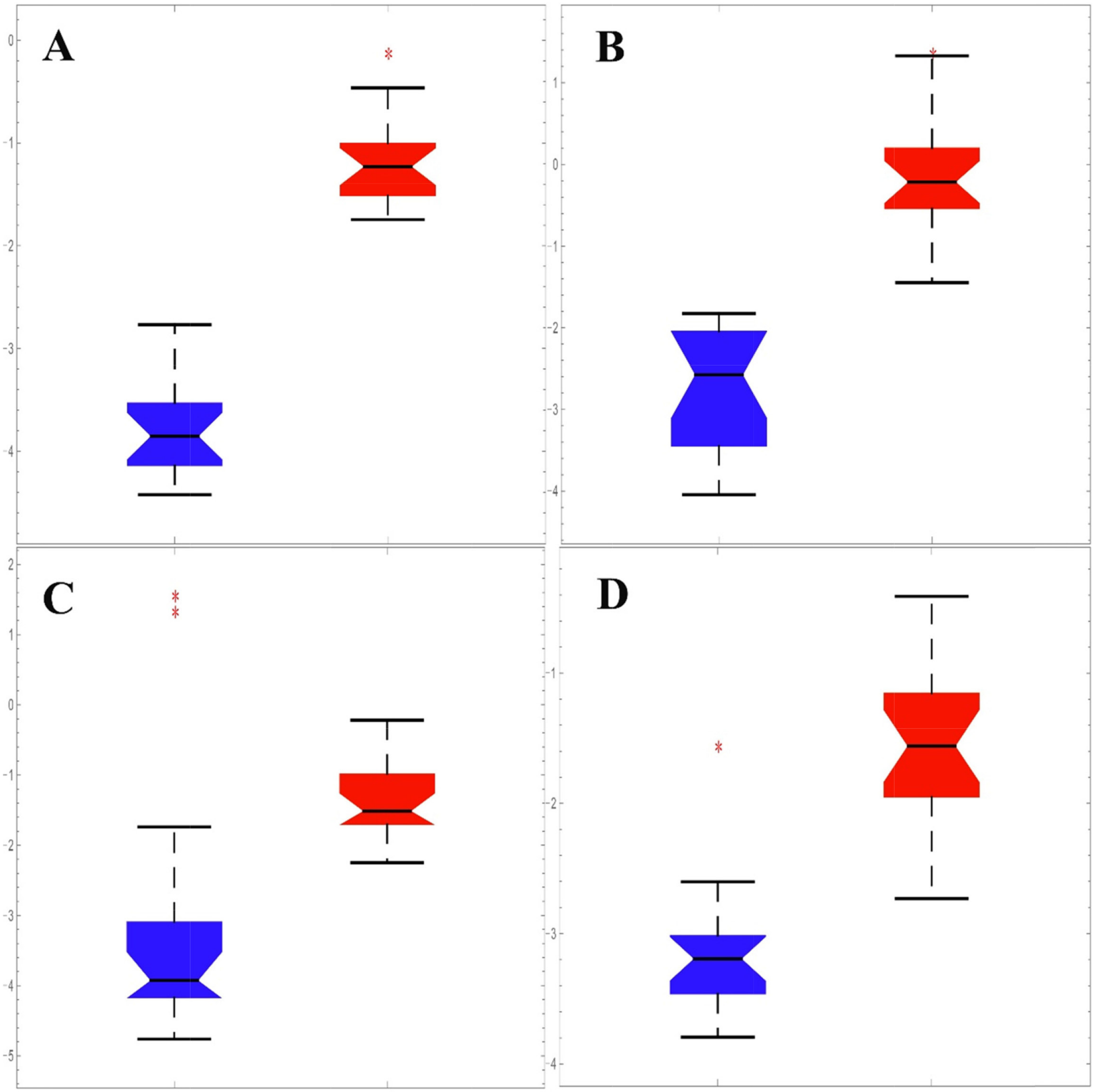

Supplementary Figure 8: The expression level of four important candidate biomarkers in tumor tissue (blue) and normal (red) was consistent with discovery stage (validation dataset GSE38241). **(A)** The expression level of SPOCK3 in GSE38241. **(B)** The expression level of SPON1 in GSE38241. **(C)** The expression level of PTN in GSE38241. **(D)** The expression level of TGFB3 in GSE38241.

Supplementary Table 1: List of prostate cancer patients related datasets used in this study

Dataset	Institute	platform	year	PMID
GSE26910	University of Turin Molecular Biotechnology and Health Sciences	Affymetrix Human Genome U133 Plus 2.0 Array	2011/03/31	21611158 [1]
GSE32448	CPDR USU	Affymetrix Human Genome U133 Plus 2.0 Array	2011/09/29	22343836 [2]
GSE46602	Aarhus University Hospital Dept. of Urology	Affymetrix Human Genome U133 Plus 2.0 Array	2015/07/01	26522007 [3]
GSE55945	Beth Israel Deaconess Medical Center Dept. of Surgery	Affymetrix Human Genome U133 Plus 2.0 Array	2014/03/18	19737960 [4]
TCGA. PRAD. sample Map/ HiSeqV2	University of North Carolina TCGA genome characterization center	IlluminaHiSeq_RNASeqV2	2016/08/16	non-available
GSE17906	Universidade de São Paulo Genetics, USP Medical School at Ribeirao Preto	Affymetrix Human Genome U133 Plus 2.0 Array	2009/09/09	19737398 [5]
GSE6919	The Methodist Hospital	Affymetrix Human Genome U95 Version 2 Array	2007/01/30	17430594 [6]
GSE38241	Massachusetts General Hospital	Agilent-014850 Whole Human Genome Microarray 4x44K G4112F	2012/05/25	23345608 [7]

Supplementary Table 2: Clinic information of prostate cancer patients

	Discovery cohort	Validation cohort
Sample size	94	78
Age		
≤65	27	-
>65	15	-
others	52	78
Gleason score		
6~7	68	49
8~10	26	21
others	-	8
PSA value		
>0.1	36	-
≤0.1	0	-
others	58	78
Pathology stage		
T1-T2	22	29
T3-T4	20	41
others	52	8

*Others means that related information were missing in the dataset.

Supplementary Table 3: List of found candidate biomarker genes in this study

Type	Gene symbol
New	SPOCK3, RNASE4, COL13A1, VWA5A, SNX21, TCEAL2, KLHL15, ARSJ, TTC28, LINC00623, NIPAL3, SPON1, MIR1908, KCNK3, FAXDC2, ANKRD35, NARR, ADGRL2
Reported	PTN, EFEMP1, EPAS1, ID4, TGFB3, ANGPT1, PENK, OCLN, TNFAIP8, ALAD, LPIN1, ADIRF, JUP, ABCC6, PLAGL1, PTGDS, GSTM3, FOXQ1, CRISPLD2, PRKG1, GSTM1, EFS, HOXD10, TLR3, TNFRSF10D

Supplementary Table 4: List of the target genes of MIR1908

MIR	Gene symbol
MIR1908-5P	ABO, ACSL3, ADAR, ADCYAP1R1, AK3, AKAP3, AP5Z1, ARAF, ARHGAP31, ARHGAP39, ARHGAP6, ARID3B, ASB16, ASH1L, BCORL1, C7orf26, CACNA2D2, CALR, CCDC169-SOHLH2-SOHLH2, CDK5R2, CIAO1, CCDC67, CDKN2A, CLSTN1, CMIP, CNN2, CNNM4, CSNK2A1, CTDSP1, CYP2B6, CYP8B1, CYTH1, CYTH2, DBN1, DPF1, DPP9, DUOXA2, DYRK1A, EFNA5, ENTPD2, EPB41L1, ESPN, ETV6, FAM73B, FBXW5, FGFRL1, FKBP8, FLT1, GBP5, GIGYF1, GMDS, GNAI2, GREM2, GRIN2D, GYS1, HOXC11, HSPG2, IGF2-INS-IGF2, IGHMBP2, ILK, IQSEC2, KIF21B, KMT2D, LDOC1, LOC100507547-PRRT1, LRRK1, LSP1, MAFB, MLLT6, MTRF1L, MVB12A, MYO1D, NAV1, NFIC, NFIX, NISCH, NR5A1, NRARP, NTN5, NUB1, OLFM2, OLIG1, OTUB1, PABPC1L2B, PCDHA3, PDGFRA, PCDHA9, PCDHAC1, PEX26, PFAS, PIK3R5, PIM3, PIP5K1C, PITPNM2, POLM, PORCN, PPFIA3, PPP5C, PRSS22, RAB35, RAP1GAP2, RFNG, RGS5, SAMD4A, SBF1, SCN1B, SLC17A7, SLC25A40, SLC25A44, SLITRK1, SOX1, SP2, SPI1, SPRY4, SPTBN4, SREBF2, STK35, STX1A, TBC1D30, TBX1, TMBIM1, TNFRSF8, TUBA8, TVP23A, UBE2M, UBE2NL, UBXN6, WIPF3, WWOX, YIF1B, ZBTB22, ZFR2, ZNF385A, ZNF516, ZNF628, ZNF784, ZNF787
MIR1908-3P	ELOVL2, NBEAL2, MURC, ACKR2, SETD1B, VGLL4, ZIC4, ANO8, MTA1
